# Insights into mucosal associated invariant T cell biology from human inborn errors of immunity

**DOI:** 10.3389/fimmu.2022.1107609

**Published:** 2022-12-22

**Authors:** Lauren J. Howson, Vanessa L. Bryant

**Affiliations:** ^1^ Immunology Division, Walter & Eliza Hall Institute of Medical Research, Melbourne, VIC, Australia; ^2^ Department of Medical Biology, The University of Melbourne, Melbourne, VIC, Australia; ^3^ Department of Clinical Immunology & Allergy, Royal Melbourne Hospital, Melbourne, VIC, Australia

**Keywords:** MAIT cells, immunodeficiency - primary, autoimmunity, immunogenetics, infection, inborn errors of immunity

## 1 Introduction

Inborn errors of immunity (IEI) are a group of inherited disorders caused by damaging variants in genes essential for immunity. Cases in which a single gene causes disease provides fundamental insights into how a single protein’s function directly impacts specific components of the immune system. Patients with IEI may present clinically with primary immunodeficiency, autoinflammation, autoimmunity and/or malignancy. IEI research is a rapidly growing field, with the recent advances in genome sequencing leading to 485 currently known monogenetic defects that cause IEI (1).

Mucosal associated invariant T (MAIT) cells are a subset of unconventional T cells that are activated following engagement of their T cell receptor (TCR) with MR1, a major histocompatibility complex (MHC) class I-related molecule that presents vitamin B metabolite antigens (2). However, MAIT cells can also be activated in a TCR-independent manner *via* cytokines, namely interleukin (IL)-12 and IL-18 (3). MAIT cell effector responses mirror conventional T-helper (Th)1 and Th17 cytokine profiles (4), but can also engage in CD8 T cell-like cytotoxic responses *via* release of granzymes and perforin (5). Due to this broad activation and effector function potential, MAIT cells have been implicated as key immune players in defense against a range of bacterial and viral infections, in addition to a role in autoimmunity and cancer (6). Despite these insights, the proteins and cells essential to support MAIT cell frequency and function, and the implications for human immunity in the context of dysfunctional MAIT cells, are only just beginning to be uncovered. Recent reports of IEI that include MAIT cell immunophenotyping, and to a limited extent functional analysis, provide an ideal opportunity to discover the fundamental factors that govern MAIT cell biology.

## 2 IEI with disruptions to MAIT cell compartment

**Figure 1:**
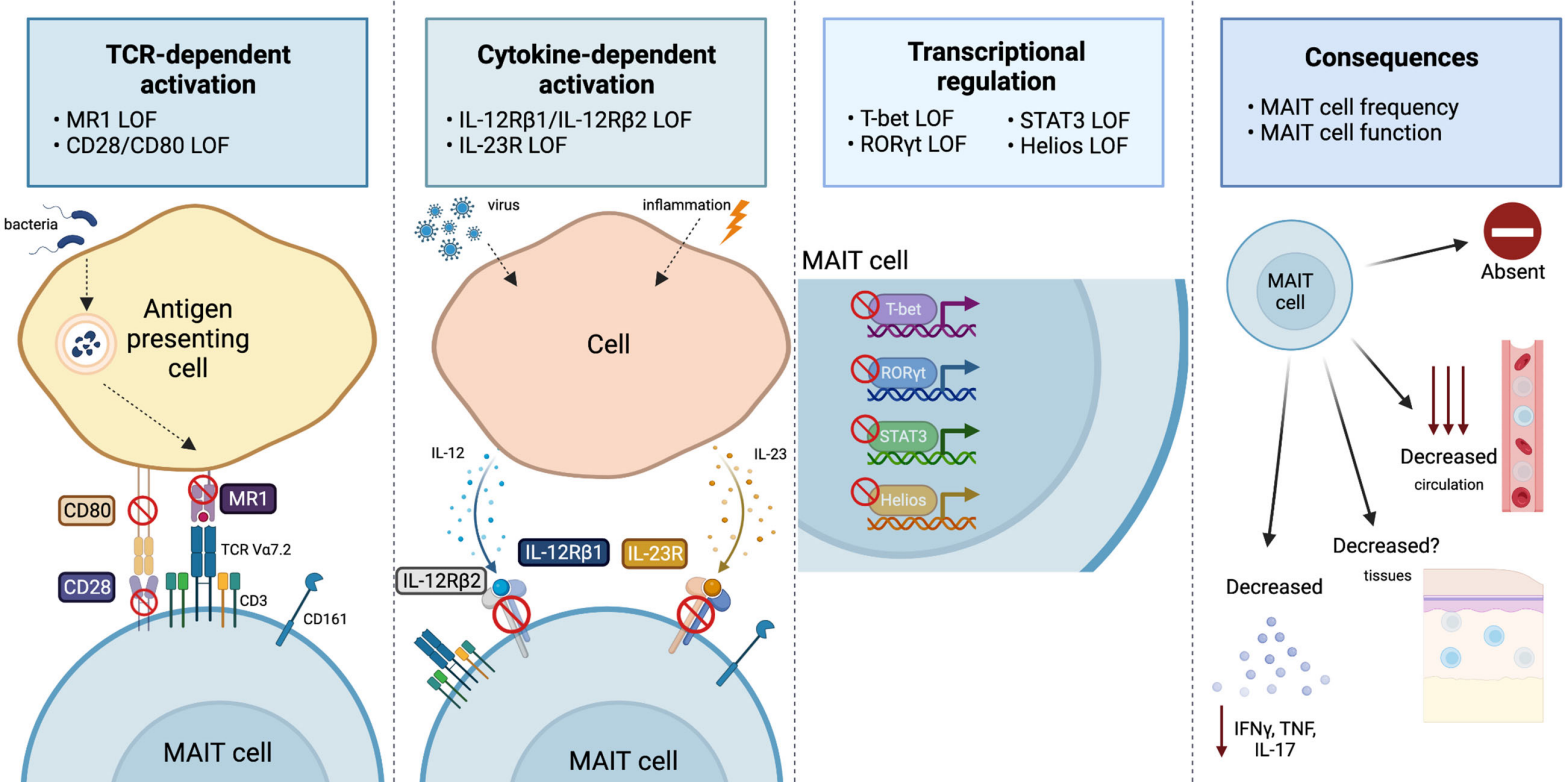
Overview of the range and consequences of MAIT cell activation and signaling pathways disrupted by IEI. MAIT cells can be stimulated *via* TCR-dependent activation, where microbial-derived vitamin B metabolites are presented on MR1 and recognized by the MAIT cell TCR Vα7.2. Disruptions to MR1 or costimulatory molecules (CD28/CD80) has been shown to impact the MAIT cell compartment. MAIT cells are also activated by viral or inflammatory conditions in which cells produce IL-12 or IL-23 in response. Cases in which IL-12Rβ1, IL-12Rβ2, or IL-23R are deficient alters the MAIT cells compartment. The transcription factors T-bet, RORγT, STAT3 and Helios all play a vital role for MAIT cell development and/or effector function, and cases in which they are deficient report alterations to the MAIT cell compartment. Ultimately, all these pathway disruptions can cause varying consequences to the MAIT cell compartment, that includes: an absence or reduction in MAIT cells in circulation (it is unknown whether this is also the case at tissue sites) or a reduction in pro-inflammatory cytokine production (IFNγ, TNF, or IL-17). Figure created with BioRender.com.

Here, we present a curated review of IEI in which MAIT cells have been assessed for frequency, phenotype and/or function (**Table 1**). The most striking disruptions reported in IEI are cases that report a complete absence of MAIT cells (**Figure 1**). Complete MAIT cell deficiency, along with an expansion of γδ T cells was observed in an individual with MR1 deficiency (29). This was the result of a homozygous point mutation in the antigen binding groove of MR1, rendering it unable to present antigen. This resulted in an immune system with a selective loss of MAIT cells. This individual’s infection history included Varicella zoster viral infection (complicated by secondary bacterial pneumonia and subsequent lung scarring) prolonged *Campylobacter* gastroenteritis with haematochezia (which was initially refractory to treatment), and extensive human papilloma virus (HPV)^+^ warts refractory to treatment. This case provided direct evidence for the importance of MAIT cells’ antigen-dependent role in controlling human bacterial infections, but also highlighted their antigen-independent role in controlling human viral infections, as had been suggested by previous mouse model (45) and observational human studies (46, 47).

**Table 1:** Summary of inborn errors of immunity that have assessed MAIT cell frequency and/or function.

Gene	Inheritance	Variant type	Gene function	Clinical presentation	Adult/pediatric	Cohort	MAIT cell frequency	MAIT cells defined by	MAIT cell phenotype	MAIT cell function	Other immune features	Ref
*ADA2*	Recessive	Loss-of-function	Enzyme (adenosine deaminase)	Autoinflammatory and immunodeficiency	Both	10	Decreased (circulating)	Surrogate markers (CD3^+^CD161^+^Vα7.2^+^)	ND	ND	↓ Tregs, Vδ2, NKT, memory B, CD4^+^ and CD8^+^ memory T cells	(7)
*AIRE*	Recessive	Loss-of-function	Autoimmune regulator	**APECED**	Both	8	Decreased (circulating)	Surrogate markers (CD3^+^CD161^+^Vα7.2^+^)	ND	ND	Neutralizing autoantibodies against type I IFN and IL-22	(8)
*BCL10*	Recessive	Loss-of-function	TCR signaling	**CID**: respirators infections	Pediatric	1	Decreased (circulating)	Surrogate markers (CD3^+^CD161^+^Vα7.2^+^)	ND	ND	Absent memory B and T cells ↓ Tregs, NK, γδ T, and Tfh cells	(9)
*BTK*	X-linked	Loss-of-function	Cell signaling (B cell)	**XLA**: bacterial infections, giardia, mycoplasma, and enteroviruses	Not provided	4	Decreased (circulating)	TRAV1-2 transcript	ND	ND	Absent circulating B cells ↓/absent serum Ig.	(10)
*CARMIL2*	Recessive	Loss-of-function	Capping protein (cell structure and migration)	**CID**: bacterial, fungal, mycobacterial infections, viral warts, molluscum, and malignancy	Both	6	Decreased (circulating)	Surrogate markers (CD3^+^CD161^+^Vα7.2^+^)	ND	ND	↑ naïve T cells ↓ Treg and memory B cells	(11)
*CD27*	Recessive	Loss-of-function	Costimulatory molecule	EBV and lymphoproliferative conditions	Both	10	Decreased (circulating)	Surrogate markers (CD3^+^CD161^+^Vα7.2^+^)	ND	ND	↑ CD8 T cells absent memory B cells	(12)
*CD28*	Recessive	Loss-of-function	Costimulatory molecule	HPV-2 and HPV-4 driven by EV	Both	3	Decreased (circulating)	Surrogate markers (CD3^+^CD161^+^Vα7.2^+^)	ND	ND	↑ naïve CD4^+^ T cells ↓ T_CM_ cells and Tregs	(13)
*CD70*	Recessive	Loss-of-function	Costimulatory ligand	EBV and lymphoproliferative conditions	Both	7	Decreased (circulating)	Surrogate markers (CD3^+^CD161^+^Vα7.2^+^)	ND	ND	↑ γδ T cells ↓ memory B cells	(12)
*CDC42*	Dominant	Loss-of-function	GTP/GDP-binding protein (actin cytoskeleton)	**Takenouchi Kosaki syndrome**	Adult	1	Within normal range	Surrogate markers (CD3^+^CD161^+^Vα7.2^+^)	ND	ND	↑ memory T and naïve B cells ↓ B and NK cells	(14)
*CFTR*	Recessive	Loss-of-function	Chloride channel	**Cystic fibrosis**	Adult	41	Decreased (circulating)	Surrogate markers (CD3^+^CD161^+^Vα7.2^+^)	ND	ND	↑ γδ T cells ↓ NK cells	(15)
*CORO1A*	Recessive	Loss-of-function	actin-regulating protein	**SCID** (leaky)	Pediatric	1	Decreased (circulating)	Surrogate markers (CD3^+^CD161^+^Vα7.2^+^)	ND	ND	↓ naive T and NKT cells	(16)
*CTPS1*	Recessive	Loss-of-function	DNA/RNA synthesis enzyme	Severe bacterial and viral infections	Pediatric	7	Decreased (circulating)	MR1 tetramer + surrogate markers (CD3^+^CD161^+^Vα7.2^+^)	ND	ND	↓ NKT, memory B and NK cells	(17)
*DOCK8*	Recessive	Loss-of-function	Guanine nucleotide exchange factor (cytoskeleton organization)	**CID:** recurrent viral, bacterial, and fungal infections, severe eczema, allergies, malignancy and autoimmunity	Both	7	Decreased (circulating)	Surrogate markers (CD3^+^CD161^+^Vα7.2^+^)	ND	ND	↓ Tregs, total T, NKT and memory B cells ↑ total B cells ↓ IgM ↑ IgG, IgA and IgE	(18)
*GATA2*	Dominant	Loss-of-function	Transcription factor (hematopoiesis)	Complex disorder of hematopoiesis with variable extramedullary defects and myelodysplasia	Both	4	Decreased (circulating)	Surrogate markers CD8^+^ CD161^+^ Va7.2^+^	ND	ND	↓ monocytes, DC, B and NK cells	(19)
*GINS1*	Recessive	(partial) Loss-of-function	DNA replication	craniofacial abnormalities, viral infections	Both	3	Decreased (circulating)	Surrogate markers (CD3^+^CD161^+^Vα7.2^+^)	ND	ND	↓ NK cells and neutrophils ↑ IgA ↓ IgM and IgG	(20)
*IFNG*	Recessive	Loss-of-function	Cytokine	**MSMD**	Pediatric	2	Within normal range	Surrogate markers (CD3^+^CD161^+^Vα7.2^+^)	ND	ND	↑ naive T cells ↓ NKT and CD27^+^ memory B cells	(21)
*IKZF2*	Dominant	Loss-of-function	Transcription factor (hematopoietic-specific)	**CID**: respiratory infections, thrush and mucosal ulcers, and chronic lymphadenopathy	Adult	2	Decreased (circulating and intestinal mucosa)	MR1 tetramer + surrogate markers (CD3^+^CD161^+^Vα7.2^+^)	High CD69	ND	↑ activated T cells ↓ naïve CD8^+^ T cells ↓ IgG	(22)
	Recessive	Loss-of-function		**CID**: sinusitis, otitis media, lower respiratory tract infections, pneumonia	Adult	1	Absent	Surrogate markers (CD3^+^CD161^+^Vα7.2^+^)	N/A	N/A	↑ γδ ↓ CD4 T, B and NK cells Absent NKT ↓ IgG	(23)
*IL12RB1*	Recessive	Loss-of-function	Cytokine receptor	**MSMD**	Not provided	4	Decreased (circulating)	Surrogate markers (CD3^+^CD161^+^Vα7.2^+^)	ND	ND	↑ naïve T cells ↓ Th1 and Th17 cells	(24)
*IL12RB2*	Recessive	Loss-of-function	Cytokine receptor	**MSMD**	Pediatric	1	Decreased (circulating)	Surrogate markers (CD3^+^CD161^+^Vα7.2^+^)	ND	ND	↑ naïve T cells ↓ Th1 cells	(25)
*IL21R*	Recessive	Loss-of-function	Cytokine receptor	**CID:** cryptosporidium infections	Both	8	Decreased (circulating)	Surrogate markers (CD3^+^CD161^+^Vα7.2^+^)	ND	ND	↓ CD4^+^ T, cTfh, memory B, NK and myeloid-derived DC ↓ IgG	(26)
*IL23R*	Recessive	Loss-of-function	Cytokine receptor	**MSMD**	Pediatric	1	Decreased (circulating)	Surrogate markers (CD3^+^CD161^+^Vα7.2^+^)	ND	ND	↑ naïve T cells ↓ Th1 cells	(25)
*IL6ST*	Recessive	Loss-of-function	Cytokine receptor	**Hyper IgE Syndrome:** staphylococcal lesions, candidiasis, severe allergy	Both	12	Decreased (circulating)	Surrogate markers (CD3^+^CD161^+^Vα7.2^+^)	ND	ND	↑ naïve T cells ↓ T_CM_, CD8^+^ T_EM_, and Tfh cells	(27)
*MAGT1*	X-linked	Loss-of-function	Magnesium transporter	**XMEN**: EBV infection, lymphoma, viral infections, respiratory and GI infections	Both	2	Decreased (circulating)	Not defined	ND	ND	↓ CD4 T, memory B, and NKT cells ↓ IgG	(28)
*MR1*	Recessive	Loss-of-function	Metabolite antigen presentation	HPV warts, difficult to treat bacterial and viral infections	Adult	1	Absent	MR1 tetramer + surrogate markers (CD3^+^CD161^+^Vα7.2^+^)	N/A	N/A	↑ Vδ2^+^ cells	(29)
*NFKB1*	Dominant	Loss-of-function	Transcription factor (NF-κB family)	**CID**: *Mycobacterium genavense* infection	Pediatric	1	Decreased (circulating)	Surrogate markers (CD3^+^TCRαβ^+^Vα7.2^+^CD161^+^)	ND	ND	↓ CD4 T, B, γδ T and NK cells ↓ IgG	(30)
*NFKB2*	Dominant	Loss-of-function	Transcription factor (NF-κB family)	Respiratory infections, pituitary dysfunction, and autoimmunity	Pediatric	1	Decreased (circulating)	Surrogate markers (CD161^+^Va7.2^+^CD8^+^)	ND	ND	Disturbed B cell differentiation ↓ IgG ↓ Lymphocyte subsets	(31)
*PDCD1*	Recessive	Loss-of-function	Immune-inhibitory receptor	Tuberculosis, autoimmunity, and hepatosplenomegaly	Pediatric	1	Decreased (circulating)	MR1 tetramer + surrogate markers (CD3^+^CD161^+^Vα7.2^+^)	ND	↓ IFN-γ production	↑ CD4^−^CD8^−^ T cells ↓ Vδ2^+^ and CD56^hi^ NK cells	(32)
*RASGRP1*	Recessive	Loss-of-function	Enzyme (catalyzes UTP to CTP)	EBV and lymphoproliferative conditions	Pediatric	1	Decreased (circulating)	Surrogate markers (CD3^+^CD161^+^Vα7.2^+^)	ND	ND	↓ B, naïve CD4^+^ and CD8^+^ T, NK cells Absence of iNKT cells	(33)
*REL*	Recessive	Loss-of-function	Transcription factor (NF-κB family)	**CID**: severe viral, bacterial, fungal, and parasitic diseases	Pediatric	1	Increased (circulating)	Surrogate markers (CD3^+^CD161^+^Vα7.2^+^)	ND	Normal IFN-γ production	↑ Vδ1^+^ and ILC2 cells ↓ Tregs and NK cells	(34)
*RORC*	Recessive	Loss-of-function	Transcription factor (nuclear hormone receptor)	Candidiasis and mycobacteriosis	Pediatric	7	Absent	MR1 tetramer + surrogate markers (CD3^+^CD161^+^Vα7.2^+^)	N/A	N/A	Absent IL-17A/F-producing T cells (including NKT cells)	(35)
*SAP*	X-linked	Loss-of-function	Signaling adaptor molecule	**XLP syndrome:** lymphohystiocytosis and lymphomas	Both	5	Within normal range	Surrogate markers (CD3^+^CD161^+^Vα7.2^+^)	ND	ND	↓ NKT cells ↓ IgG	(36)
*SASH3*	X-linked	Loss-of-function	Adaptor protein (cell signaling)	**CID**: infections and refractory autoimmune cytopenias	Adult	4	Decreased (circulating)	Surrogate markers (CD3^+^CD161^+^Vα7.2^+^)	ND	ND	↓ CD4^+^ T and NK cells	(37)
*SH2D1A*	X-linked	Loss-of-function	SLAM associated protein (SAP, signaling)	Susceptibility to EBV and lymphoproliferative conditions	Not provided	5	Within normal range	Surrogate markers (CD3^+^CD161^+^Vα7.2^+^)	Normal ZBTB16 levels	ND	↓ NKT, memory B and NK cells	(10)
*SPPL2A*	Recessive	Loss-of-function	Transmembrane protease	**MSMD**	Pediatric	3	Within normal range	Not defined	ND	ND	Absence of cDC2 cells	(38)
*STAT3*	Dominant	Loss-of-function	Transcription factor (gene regulation)	**Hyper IgE Syndrome:** craniofacial abnormalities, bacterial infections, eczema, candidiasis, osteoporosis, coronary and cerebral aneurysms	Not provided	23	Decreased (circulating)	MR1 tetramer + surrogate markers (CD3^+^CD161^+^Vα7.2^+^)	Normal RORγt and PLZF expression	↓ IL-17A and IL-17F but normal IFNγ and TNF production	↓ Th17, Tfh, NKT and memory B cells ↑ IgE	(24)
*STIM1*	Recessive	(partial) Loss-of-function	Ca2^+^-sensing	**CID:** late onset with inflammatory manifestations (psoriasis and colitis)	Both	2	Decreased (circulating)	Surrogate markers (CD3^+^CD161^+^Vα7.2^+^)	ND	ND	NKT cells absent	(39)
*TBX21*	Recessive	Loss-of-function	Transcription factor (lineage-defining)	**MSMD**	Pediatric	1	Decreased (circulating)	MR1 tetramer + surrogate markers (CD3^+^CD161^+^Vα7.2^+^)	ND	Impaired IFNγ production	↓ CD4^+^ T, iNKT, Vδ2^+^ and NK cells	(40)
*USP18*	Recessive	Loss-of-function (partial)	Negative regulator of type I IFN signaling	type I interferonopathy: autoinflammation and mycobacterial disease	Adult	1	Decreased (circulating)	Surrogate markers (CD3^+^CD161^+^Vα7.2^+^)	ND	ND	Impaired IL-12/IL-23 production by myeloid cells	(41)
*XIAP*	X-linked	Loss-of-function	Inhibitor-of-apoptosis protein	**XLP syndrome:** lymphohystiocytosis and lymphomas	Both	16	Decreased (circulating)	Surrogate markers (CD3^+^CD161^+^Vα7.2^+^)	ND	↑ apoptosis after stimulation	↓ IgG ↓ NKT cells	(36)
*ZAP70*	Recessive	Loss-of-function	Protein tyrosine kinase (TCR signaling)	**CID:** infant onset with severe infections caused by varicella zoster virus and live vaccines	Pediatric	1	Absent	Surrogate markers (CD3^+^CD161^+^Vα7.2^+^)	N/A	N/A	↓ CD8^+^ T cells NKT cells absent	(42)
*ZNF341*	Recessive	Loss-of-function	Transcription factor (STAT signaling)	**Hyper IgE syndrome:** candidiasis, staphylococcal infections, severe allergy	Both	6	Decreased (circulating)	Surrogate markers (CD3^+^CD161^+^Vα7.2^+^)	ND	ND	↑ naïve CD4^+^ T cells ↓ T_CM_, memory B, ILC1, ILC2 and NK cells	(43)
*ZNFX1*	Recessive	Loss-of-function	Helicase	Mycobacterial disease	Both	3	Within normal range	Surrogate markers (CD3^+^CD161^+^Vα7.2^+^)	ND	Normal IFN-γ production	↓ NK cells	(44)

APECED, autoimmune polyendocrinopathy candidiasis ectodermal dystrophy; CID, combined immunodeficiency; DC, dendritic cell; EBV, Epstein–Barr virus; EV, epidermodysplasia verruciformis; HPV, human papillomavirus; IFN, interferon; IL, interleukin; ILC, innate lymphoid cell; MAIT, mucosal-associated invariant T; MSMD, mendelian susceptibility to mycobacterial diseases; N/A, not applicable; ND, not determined; NK, natural killer; SCID, severe combined immunodeficiency; TCM, T central memory; TCR, T cell receptor; TEM, T effector memory; Tfh, T follicular helper; Tregs, regulatory T cell; XLA, X-linked agammaglobulinemia; XLP, X-linked lymphoproliferative; XMEN, X-linked immunodeficiency with magnesium defect, Epstein-Barr virus infection, and neoplasia. Clinical presentations in bold indicate name of disease/disorder.

Absence of MAIT cells was also reported in seven individuals with RORγT deficiency (35), along with a lack of Th17 and natural killer T (NKT) cells in these patients, who presented with common features of candidiasis and mycobacterial disease. MAIT cells were also reportedly absent in a ZAP70-deficient patient who initially presented with CD8^+^ T cell lymphopenia and severe viral infections (42). These examples highlight the exceptionally rare instances of individuals with a deficiency of a protein essential for either MAIT cell development or peripheral maintenance. The immunological phenotype and clinical presentation of those with a MAIT cell deficiency were varied, but all involved disturbances to the T cell compartment and frequent, severe, or difficult to treat infections.

By far the most common observation reported across IEI describe a decrease in the proportion (or total number) of circulating MAIT cells. Reduced frequencies of circulating MAIT cells have been reported for a range of different IEI that have a diverse clinical and/or immunological presentation, including: combined immunodeficiency (CID), X-linked agammaglobulinemia (XLA), Mendelian susceptibility to mycobacterial diseases (MSMD), X-linked immunodeficiency with magnesium defect, Epstein-Barr virus infection, and neoplasia (XMEN), and X-linked lymphoproliferative (XLP) syndrome. The majority of which are characterized by altered T and/or B cell compartments. Genes with variants related to a decrease in MAIT cells can range from: costimulatory receptors (e.g. *CD28*) (12, 13), cell structure proteins (e.g. *CARMIL2/RLTPR*) (11, 16, 18), cytokine receptors (e.g. *IL12RB1/IL12RB2*) (24–27), DNA replication proteins (e.g. *GINS1*) (17, 20) and transcription factors (e.g. *TBX21*) (19, 22, 30, 31, 40, 43) (see **Table 1** for full list).

Interestingly, a single case report described an expansion of MAIT cells in a child with c-Rel deficiency presenting with a history of severe viral, bacterial, fungal, and parasitic infections (34). Vδ1 and innate-lymphoid cells (ILC) were also expanded, and reduced frequencies of natural killer (NK) and regulatory T cells (Tregs), compared to pediatric healthy controls. However, with only a single case, it is difficult to interpret whether this MAIT cell expansion is attributable to the specific IEI, or simply individual variation. Of the IEI studies that measured and reported MAIT cell frequency, six have described frequencies of MAIT cells within a normal range in their patient cohorts (10, 14, 21, 36, 38, 44). Together, these reports demonstrate that reduced frequency of MAIT cells is a common, but not a universal, observation in IEI.

MAIT cell frequency is also impacted by loss-of-function variants in *IKZF2*, which encodes the T cell transcriptional regulator Helios. Helios deficiency can present as dominant or recessive CID with varying severity. A heterozygous *IKZF2* variant was reported in a proband and her father presenting with mild CID characterized by recurrent upper respiratory infections, mucosal ulcers, and chronic lymphadenopathy (22). The immune phenotype was chronic activation and proinflammatory cytokine production by both effector and regulatory T cells, but immune subset frequencies largely remained intact. A homozygous *IKZF2* variant in a single case presented with a more severe CID characterized by recurrent lower respiratory tract infections, leading to multiple pneumonias requiring hospitalization (23). The immune phenotype was more pronounced, with reductions in: CD4^+^ T, B, and NK cells and an absence of NKT cells. Even with differing presentations, both studies reported a decrease or absence of MAIT cells due to the *IKZF2* variants. Together, this demonstrates that MAIT cells are particularly susceptible to changes in Helios function, compared to other immune cell subsets.

The Helios deficiency study by Hetemäki et al. (22) extended beyond the typical circulating MAIT cell enumeration to measure tissue resident MAIT cells. MAIT cells are mucosal associated as their name suggests, with a large proportion populating mucosal sites. It is not well understood whether the MAIT cell circulating frequency reflects that of their tissue-associated counterparts. Colon and duodenal biopsies were examined from two individuals with Helios deficiency and a decrease in MAIT cell frequency was observed in all tissues examined when compared to healthy donor tissue (22). Therefore, this reduction in tissue associated MAIT cells suggests a global decrease of MAIT cells, rather than a redistribution to the tissues.

Establishing whether reported changes in frequency are due directly to the inborn error itself, a result of a secondary effect of the IEI on other immune components that interact with MAIT cells, or simply the result of MAIT cells responding to a clinical history of repeated episodes of prolonged infection and inflammation, is challenging. Thus, we will next discuss the strict considerations for reporting on MAIT cells in the context of IEI.

## 3 Considerations for MAIT cell frequency reporting for individuals with IEI

There is no standardized method for reporting MAIT cell frequency, with variation in how they are reported and how they are identified/defined. The most common (and accessible) method for MAIT cell identification is *via* expression of surrogate surface markers: TCR-Vα7.2 and CD161 (10). However, it is more accurate to define MAIT cells using MR1 tetramers loaded with 5-OP-RU (48). TCR-Vα7.2^+^ and CD161^++^ cells overlap 96% with MR1-tetramer^+^ cells in circulation. While it is an appropriate method for identifying MAIT cells (49), it is important to consider if the IEI impacts expression of CD161, in which case MR1 tetramers should be used for identification instead.

A major issue in reporting circulating MAIT cell frequency in humans is that no standardized frequency or number values have been established. Also, MAIT cell markers/tetramers are not typically included in standard clinical T cell panels. Thus, studies either establish their own standard values, with reference to an internal healthy control group, or patient values are compared to the typical 1–5% of circulating T cells reference range set by earlier studies of MAIT cells (4, 10, 49). Importantly, this simplified reference range does not consider variation of circulating MAIT cells present in different healthy control populations. Previous studies report MAIT cell frequency is significantly impacted by both age and sex (50, 51). MAIT cells steadily increase and peak at 20–29 years of age, before progressively declining during aging (49). Thus, it is important for IEI studies to compare to an age-matched MAIT cell value for each patient, either internally or referencing external age-matched values, to confidently report any alterations to normal frequency.

Another consideration when interpreting data on MAIT cells is the infection status of the patient at the time of analysis. MAIT cells have been shown to dynamically change in frequency during an acute infection (52) with studies in mice suggesting they accumulate and expand at the tissue site of infection (45, 53). Thus, an active infection could cause a decrease in circulating MAIT cells that may be unrelated to the underlying genetic defect. In addition, the use of corticosteroids to treat asthma and chronic obstructive pulmonary disease have also been shown to impact MAIT cell frequency (54, 55). Therefore, detailed clinical history, list of current medications, and the current infection status at the time of sampling should be provided. A more definitive approach to assess circulating MAIT cell frequency is to measure multiple samples over time. This would provide important insight into the stability of any observed change in MAIT cell frequency. This approach would also control for any infection-induced fluctuations when functionally assessing T cell (including MAIT cell) activation, proliferation, and cytotoxicity markers. As these would also be expected to alter with varying infection status.

Examination of tissue biopsies (particularly from areas of inflammation or infection), although challenging to obtain, would also address the question of MAIT cell kinetics in IEI. By directly examining MAIT cell frequency at tissue sites, it could then be correlated back to the proportion of circulating cells. This would provide an understanding of the relationship between circulating and tissue-resident MAIT cell populations, and if disturbances in MAIT cell frequency are directly attributable to the underlying IEI, rather than a consequence of increased inflammation/infection-induced tissue-homing.

## 4 Limited MAIT cell functional analysis in IEI

A less explored aspect of MAIT cells in IEI is the potential changes in their ability to respond to stimuli. MAIT cells can be activated *via* TCR-dependent or TCR-independent stimulation (2, 3). Factors that control or influence these separate activation pathways in MAIT cells could be elucidated by studying the functional response of MAIT cells from IEI patients.

Several studies have examined interferon (IFN)γ production by MAIT cells in IEI. MAIT cells from a PD-1 deficient patient produced less IFNγ in response to bacille Calmette-Guérin (BCG) + IL-12 stimulation (32). Also, MAIT cells from a T-bet deficient patient produced less IFNγ in response to phorbol myristate acetate (PMA)/ionomycin stimulation (40). However, in addition to IFNγ, MAIT cells can also produce proinflammatory cytokines TNF and IL-17A, as well as cytotoxic granules and perforin, that should be considered when undertaking functional analysis (4).

The most comprehensive functional analysis of MAIT cells in IEI was in individuals with STAT3 loss-of-function (n = 5–7) (24). STAT3-deficient MAIT cells produced normal levels of IFNγ, TNF and granzyme B when stimulated with PMA/ionomycin. However, they showed impaired IL-17A production under these conditions. In addition, STAT3-deficient MAIT cells were unable to produce IL-17A or IL-17F in Th17 culture conditions, suggesting a direct role for STAT3 regulating *IL17A*/*IL17F* transcription in MAIT cells. These functional results mirror what was observed for the functional dysregulation of STAT3-deficient CD4^+^ (Th17) T cells in the same individuals. These observations highlight the importance of assessing polyfunctionality of MAIT cell responses to stimuli in IEI, as it may provide fundamental insights into the key proteins required for differing MAIT cell effector functions.

## 5 Conclusion

MAIT cells are a particularly interesting immune subset to study in IEI. Given the signaling, activation, and functional pathways shared with NKT, γδ, CD8^+^ and Th17 T cells, it is not surprising that MAIT cells are often at the intersection of various immune cell effector responses across innate and adaptive immunity. However, it is important when contributing to, and assessing, the literature on MAIT cells in IEI that certain key factors are taken into consideration. It is essential to understand how MAIT cells are defined, and the comparative healthy reference ranges, to make informed interpretations of the impact of IEI on MAIT cell biology. Finally, the infection status at the time of sampling can also impact the strength of conclusions of these studies. In conclusion, MAIT cells are understudied yet play a unique role in human immunity, at the intersection of innate and adaptive responses. Understanding MAIT cells in the context of IEI provides an opportunity to understand their role and potential to contribute to immune dysregulation in IEI.

## Author contributions

LJH: conceptualization, writing - original draft preparation. VLB: conceptualization, writing - reviewing and editing. All authors contributed to the article and approved the submitted version.

## References

[1] Tangye SG Al-Herz W Bousfiha A Cunningham-Rundles C Franco JL Holland SM. Human inborn errors of immunity: 2022 update on the classification from the international union of immunological societies expert committee. J Clin Immunol (2022) 42:1473–507. doi: 10.1007/s10875-022-01289-3 35748970 PMC9244088

[2] Kjer-Nielsen L Patel O Corbett AJ Le Nours J Meehan B Liu L. Et al. MR1 presents microbial vitamin b metabolites to MAIT cells. Nature. (2012) 491(7426):717–23. doi: 10.1038/nature11605 23051753

[3] Ussher JE Bilton M Attwod E Shadwell J Richardson R de Lara C. CD161++ CD8+ T cells, including the MAIT cell subset, are specifically activated by IL-12+IL-18 in a TCR-independent manner. Eur J Immunol (2014) 44(1):195–203. doi: 10.1002/eji.201343509 24019201 PMC3947164

[4] Dusseaux M Martin E Serriari N Péguillet I Premel V Louis D. Human MAIT cells are xenobiotic-resistant, tissue-targeted, CD161hi IL-17-secreting T cells. Blood. (2011) 117(4):1250–9. doi: 10.1182/blood-2010-08-303339 21084709

[5] Kurioka A Ussher JE Cosgrove C Clough C Fergusson JR Smith K. MAIT cells are licensed through granzyme exchange to kill bacterially sensitized targets. Mucosal Immunol (2015) 8(2):429–40. doi: 10.1038/mi.2014.81 PMC4288950 25269706

[6] Howson LJ Salio M Cerundolo V. MR1-restricted mucosal-associated invariant T cells and their activation during infectious disease. Front Immunol (2015) 6:303. doi: 10.3389/fimmu.2015.00303 26136743 PMC4468870

[7] Yap JY Moens L Lin MW Kane A Kelleher A Toong C. Intrinsic defects in b cell development and differentiation, T cell exhaustion and altered unconventional T cell generation characterize human adenosine deaminase type 2 deficiency. J Clin Immunol (2021) 41(8):1915–35. doi: 10.1007/s10875-021-01141-0 PMC8604888 34657246

[8] Kaleviste E Rühlemann M Kärner J Haljasmägi L Tserel L Org E. 22 paucity in APECED is associated with mucosal and microbial alterations in oral cavity. Front Immunol (2020) 11:838. doi: 10.3389/fimmu.2020.00838 32477345 PMC7232598

[9] Garcia-Solis B Van Den Rym A Pérez-Caraballo JJ Al-Ayoubi A Alazami AM Lorenzo L. Clinical and immunological features of human BCL10 deficiency. Front Immunol (2021) 12:786572. doi: 10.3389/fimmu.2021.786572 34868072 PMC8633570

[10] Martin E Treiner E Duban L Guerri L Laude H Toly C. Stepwise development of MAIT cells in mouse and human. PloS Biol (2009) 7(3):e54. doi: 10.1371/journal.pbio.1000054 19278296 PMC2653554

[11] Wang Y Ma CS Ling Y Bousfiha A Camcioglu Y Jacquot S. Dual T cell- and b cell-intrinsic deficiency in humans with biallelic RLTPR mutations. J Exp Med (2016) 213(11):2413–35. doi: 10.1084/jem.20160576 PMC5068239 27647349

[12] Ghosh S Köstel Bal S Edwards ESJ Pillay B Jiménez Heredia R Erol Cipe F. Extended clinical and immunological phenotype and transplant outcome in CD27 and CD70 deficiency. Blood. (2020) 136(23):2638–55. doi: 10.1182/blood.2020006738 PMC7735164 32603431

[13] Béziat V Rapaport F Hu J Titeux M Bonnet des Claustres M Bourgey M. Humans with inherited T cell CD28 deficiency are susceptible to skin papillomaviruses but are otherwise healthy. Cell. (2021) 184(14):3812–28.e30. doi: 10.1016/j.cell.2021.06.004 34214472 PMC8329841

[14] Bucciol G Pillay B Casas-Martin J Delafontaine S Proesmans M Lorent N. Systemic inflammation and myelofibrosis in a patient with takenouchi-kosaki syndrome due to CDC42 Tyr64Cys mutation. J Clin Immunol (2020) 40(4):567–70. doi: 10.1007/s10875-020-00742-5 31953712

[15] Smith DJ Hill GR Bell SC Reid DW. Reduced mucosal associated invariant T-cells are associated with increased disease severity and pseudomonas aeruginosa infection in cystic fibrosis. PloS One (2014) 9(10):e109891. doi: 10.1371/journal.pone.0109891 25296025 PMC4190362

[16] Moshous D Martin E Carpentier W Lim A Callebaut I Canioni D. Whole-exome sequencing identifies coronin-1A deficiency in 3 siblings with immunodeficiency and EBV-associated b-cell lymphoproliferation. J Allergy Clin Immunol (2013) 131(6):1594–603. doi: 10.1016/j.jaci.2013.01.042 PMC3824285 23522482

[17] Martin E Minet N Boschat AC Sanquer S Sobrino S Lenoir C. Impaired lymphocyte function and differentiation in CTPS1-deficient patients result from a hypomorphic homozygous mutation. JCI Insight (2020) 5(5):e133880. doi: 10.1172/jci.insight.133880 32161190 PMC7141395

[18] Pillay BA Avery DT Smart JM Cole T Choo S Chan D. Hematopoietic stem cell transplant effectively rescues lymphocyte differentiation and function in DOCK8-deficient patients. JCI Insight (2019) 5(11):e127527. doi: 10.1172/jci.insight.127527 31021819 PMC6629099

[19] Dickinson RE Milne P Jardine L Zandi S Swierczek SI McGovern N. The evolution of cellular deficiency in GATA2 mutation. Blood. (2014) 123(6):863–74. doi: 10.1182/blood-2013-07-517151 PMC3916878 24345756

[20] Cottineau J Kottemann MC Lach FP Kang YH Vély F Deenick EK. Inherited GINS1 deficiency underlies growth retardation along with neutropenia and NK cell deficiency. J Clin Invest. (2017) 127(5):1991–2006. doi: 10.1172/JCI90727 28414293 PMC5409070

[21] Kerner G Rosain J Guérin A Al-Khabaz A Oleaga-Quintas C Rapaport F. Inherited human IFN-γ deficiency underlies mycobacterial disease. J Clin Invest. (2020) 130(6):3158–71. doi: 10.1172/JCI135460 PMC7260033 32163377

[22] Hetemäki I Kaustio M Kinnunen M Heikkilä N Keskitalo S Nowlan K. Loss-of-function mutation in IKZF2 leads to immunodeficiency with dysregulated germinal center reactions and reduction of MAIT cells. Sci Immunol (2021) 6(65):eabe3454. doi: 10.1126/sciimmunol.abe3454 34826260

[23] Shahin T Kuehn HS Shoeb MR Gawriyski L Giuliani S Repiscak P. Germline biallelic mutation affecting the transcription factor Helios causes pleiotropic defects of immunity. Sci Immunol (2021) 6(65):eabe3981. doi: 10.1126/sciimmunol.abe3981 34826259 PMC7612971

[24] Wilson RP Ives ML Rao G Lau A Payne K Kobayashi M. STAT3 is a critical cell-intrinsic regulator of human unconventional T cell numbers and function. J Exp Med (2015) 212(6):855–64. doi: 10.1084/jem.20141992 PMC4451129 25941256

[25] Martínez-Barricarte R Markle JG Ma CS Deenick EK Ramírez-Alejo N Mele F. Human IFN-γ immunity to mycobacteria is governed by both IL-12 and IL-23. Sci Immunol (2018) 3(30):eaau6759. doi: 10.1126/sciimmunol.aau6759 30578351 PMC6380365

[26] Cagdas D Mayr D Baris S Worley L Langley DB Metin A. Genomic spectrum and phenotypic heterogeneity of human IL-21 receptor deficiency. J Clin Immunol (2021) 41(6):1272–90. doi: 10.1007/s10875-021-01031-5 PMC8086229 33929673

[27] Béziat V Tavernier SJ Chen YH Ma CS Materna M Laurence A. Dominant-negative mutations in human IL6ST underlie hyper-IgE syndrome. J Exp Med (2020) 217(6):e20191804. doi: 10.1084/jem.20191804. 32207811 PMC7971136

[28] Klinken EM Gray PE Pillay B Worley L Edwards ESJ Payne K. Diversity of XMEN disease: Description of 2 novel variants and analysis of the lymphocyte phenotype. J Clin Immunol (2020) 40(2):299–309. doi: 10.1007/s10875-019-00732-2 31865525

[29] Howson LJ Awad W von Borstel A Lim HJ McWilliam HEG Sandoval-Romero ML. Absence of mucosal-associated invariant T cells in a person with a homozygous point mutation in MR1. Sci Immunol (2020) 5(49). doi: 10.1126/sciimmunol.abc9492 PMC8581949 32709702

[30] Gonzalez-Granado LI Ruiz-García R Blas-Espada J Moreno-Villares JM Germán-Diaz M López-Nevado M. Acquired and innate immunity impairment and severe disseminated mycobacterium genavense infection in a patient with a NF-κB1 deficiency. Front Immunol (2018) 9:3148. doi: 10.3389/fimmu.2018.03148 30761159 PMC6362422

[31] Klemann C Camacho-Ordonez N Yang L Eskandarian Z Rojas-Restrepo JL Frede N. Clinical and immunological phenotype of patients with primary immunodeficiency due to damaging mutations in NFKB2. Front Immunol (2019) 10:297. doi: 10.3389/fimmu.2019.00297 30941118 PMC6435015

[32] Ogishi M Yang R Aytekin C Langlais D Bourgey M Khan T. Inherited PD-1 deficiency underlies tuberculosis and autoimmunity in a child. Nat Med (2021) 27(9):1646–54. doi: 10.1038/s41591-021-01388-5 PMC8446316 34183838

[33] Winter S Martin E Boutboul D Lenoir C Boudjemaa S Petit A. Loss of RASGRP1 in humans impairs T-cell expansion leading to Epstein-Barr virus susceptibility. EMBO Mol Med (2018) 10(2):188–99. doi: 10.15252/emmm.201708292 PMC5801500 29282224

[34] Lévy R Langlais D Béziat V Rapaport F Rao G Lazarov T. Inherited human c-rel deficiency disrupts myeloid and lymphoid immunity to multiple infectious agents. J Clin Invest (2021) 131(17). doi: 10.1172/JCI150143 PMC8409595 34623332

[35] Okada S Markle JG Deenick EK Mele F Averbuch D Lagos M. IMMUNODEFICIENCIES. impairment of immunity to candida and mycobacterium in humans with bi-allelic RORC mutations. Science. (2015) 349(6248):606–13. doi: 10.1126/science.aaa4282 PMC4668938 26160376

[36] Rigaud S Fondanèche MC Lambert N Pasquier B Mateo V Soulas P. XIAP deficiency in humans causes an X-linked lymphoproliferative syndrome. Nature. (2006) 444(7115):110–4. doi: 10.1038/nature05257 17080092

[37] Delmonte OM Bergerson JRE Kawai T Kuehn HS McDermott DH Cortese I. SASH3 variants cause a novel form of X-linked combined immunodeficiency with immune dysregulation. Blood. (2021) 138(12):1019–33. doi: 10.1182/blood.2020008629 PMC8462359 33876203

[38] Kong XF Martinez-Barricarte R Kennedy J Mele F Lazarov T Deenick EK. Disruption of an antimycobacterial circuit between dendritic and helper T cells in human SPPL2a deficiency. Nat Immunol (2018) 19(9):973–85. doi: 10.1038/s41590-018-0178-z PMC6130844 30127434

[39] Schaballie H Rodriguez R Martin E Moens L Frans G Lenoir C. A novel hypomorphic mutation in STIM1 results in a late-onset immunodeficiency. J Allergy Clin Immunol (2015) 136(3):816–9.e4. doi: 10.1016/j.jaci.2015.03.009 25935105

[40] Yang R Mele F Worley L Langlais D Rosain J Benhsaien I. Human T-bet governs innate and innate-like adaptive IFN-γ immunity against mycobacteria. Cell. (2020) 183(7):1826–47.e31. doi: 10.1016/j.cell.2020.10.046 33296702 PMC7770098

[41] Martin-Fernandez M Buta S Le Voyer T Li Z Dynesen LT Vuillier F. A partial form of inherited human USP18 deficiency underlies infection and inflammation. J Exp Med (2022) 219(4). doi: 10.1084/jem.20211273 PMC8908790 35258551

[42] Hauck F Blumenthal B Fuchs S Lenoir C Martin E Speckmann C. SYK expression endows human ZAP70-deficient CD8 T cells with residual TCR signaling. Clin Immunol (2015) 161(2):103–9. doi: 10.1016/j.clim.2015.07.002 26187144

[43] Béziat V Li J Lin JX Ma CS Li P Bousfiha A. A recessive form of hyper-IgE syndrome by disruption of ZNF341-dependent STAT3 transcription and activity. Sci Immunol (2018) 3(24):eaat4956. doi: 10.1126/sciimmunol.aat4956 29907691 PMC6141026

[44] Le Voyer T Neehus AL Yang R Ogishi M Rosain J Alroqi F. Inherited deficiency of stress granule ZNFX1 in patients with monocytosis and mycobacterial disease. Proc Natl Acad Sci U.S.A. (2021) 118(15). doi: 10.1073/pnas.2102804118 PMC8053974 33876776

[45] van Wilgenburg B Loh L Chen Z Pediongco TJ Wang H Shi M. MAIT cells contribute to protection against lethal influenza infection in vivo. Nat Commun (2018) 9(1):4706. doi: 10.1038/s41467-018-07207-9 30413689 PMC6226485

[46] van Wilgenburg B Scherwitzl I Hutchinson EC Leng T Kurioka A Kulicke C. MAIT cells are activated during human viral infections. Nat Commun (2016) 7:11653. doi: 10.1038/ncomms11653 27337592 PMC4931007

[47] Flament H Rouland M Beaudoin L Toubal A Bertrand L Lebourgeois S. Outcome of SARS-CoV-2 infection is linked to MAIT cell activation and cytotoxicity. Nat Immunol (2021) 22(3):322–35. doi: 10.1038/s41590-021-00870-z 33531712

[48] Corbett AJ Eckle SB Birkinshaw RW Liu L Patel O Mahony J. T-Cell activation by transitory neo-antigens derived from distinct microbial pathways. Nature. (2014) 509(7500):361–5. doi: 10.1038/nature13160 24695216

[49] Gherardin NA Souter MN Koay HF Mangas KM Seemann T Stinear TP. Human blood MAIT cell subsets defined using MR1 tetramers. Immunol Cell Biol (2018) 96(5):507–25. doi: 10.1111/imcb.12021 PMC6446826 29437263

[50] Novak J Dobrovolny J Novakova L Kozak T. The decrease in number and change in phenotype of mucosal-associated invariant T cells in the elderly and differences in men and women of reproductive age. Scand J Immunol (2014) 80(4):271–5. doi: 10.1111/sji.12193 24846411

[51] Lee OJ Cho YN Kee SJ Kim MJ Jin HM Lee SJ. Circulating mucosal-associated invariant T cell levels and their cytokine levels in healthy adults. Exp Gerontol. (2014) 49:47–54. doi: 10.1016/j.exger.2013.11.003 24269212

[52] Howson LJ Napolitani G Shepherd D Ghadbane H Kurupati P Preciado-Llanes L. MAIT cell clonal expansion and TCR repertoire shaping in human volunteers challenged with salmonella paratyphi a. Nat Commun (2018) 9(1):253. doi: 10.1038/s41467-017-02540-x 29343684 PMC5772558

[53] Wang H D'Souza C Lim XY Kostenko L Pediongco TJ Eckle SBG. MAIT cells protect against pulmonary legionella longbeachae infection. Nat Commun (2018) 9(1):3350. doi: 10.1038/s41467-018-05202-8 30135490 PMC6105587

[54] Hinks TS Zhou X Staples KJ Dimitrov BD Manta A Petrossian T. Innate and adaptive T cells in asthmatic patients: Relationship to severity and disease mechanisms. J Allergy Clin Immunol (2015) 136(2):323–33. doi: 10.1016/j.jaci.2015.01.014 PMC4534770 25746968

[55] Hinks TS Wallington JC Williams AP Djukanović R Staples KJ Wilkinson TM. Steroid-induced deficiency of mucosal-associated invariant T cells in the chronic obstructive pulmonary disease lung. implications for nontypeable haemophilus influenzae infection. Am J Respir Crit Care Med (2016) 194(10):1208–18. doi: 10.1164/rccm.201601-0002OC PMC5114442 27115408

